# Helminth-derived proteins as immune system regulators: a systematic review of their promise in alleviating colitis

**DOI:** 10.1186/s12865-024-00614-2

**Published:** 2024-04-18

**Authors:** Maimonah Alghanmi, Faisal Minshawi, Tarfa A. Altorki, Ayat Zawawi, Isra Alsaady, Abdallah Y Naser, Hassan Alwafi, Soa’ad M. Alsulami, Ala A. Azhari, Anwar M Hashem, Rowa Alhabbab

**Affiliations:** 1 Department of Medical Laboratory Sciences, Faculty of Applied Medical Sciences, King Abdulaziz University, Jeddah, Saudi Arabia; 2 Department of Laboratory Medicine, Faculty of Applied Medical Sciences, Umm Al-Qura University, Makkah, Saudi Arabia; 3 Vaccines and Immunotherapy Unit, King Fahd Medical Research Center, King Abdulaziz University, Jeddah, Saudi Arabia; 4 Special Infectious Agent Unit, King Fahad Medical Research Center, King Abdulaziz University, Jeddah, Saudi Arabia; 5 Clinical and Molecular Microbiology Laboratories, King Abdulaziz University Hospital, King Abdulaziz University, Jeddah, 21589 Saudi Arabia; 6 Department of Applied Pharmaceutical Sciences and Clinical Pharmacy, Faculty of Pharmacy, Isra University, Amman, Jordan; 7 Faculty of Medicine, Umm Al-Qura University, Makkah, Saudi Arabia; 8 Department of Clinical Microbiology and Immunology, Faculty of Medicine, King Abdulaziz University, Jeddah, Saudi Arabia

**Keywords:** Helminths, Immunoregulation, Inflammatory bowel disease, Colitis, Ulcerative colitis, Crohn’s disease

## Abstract

Helminth-derived proteins have immunomodulatory properties, influencing the host’s immune response as an adaptive strategy for helminth survival. Helminth-derived proteins modulate the immune response by inducing anti-inflammatory cytokines, promoting regulatory T-cell development, and ultimately favouring a Th2-biased immune response. This systematic review focused on helminth-derived proteins and explored their impact on reducing inflammatory responses in mouse models of colitis. A systematic search across Medline, EMBASE, Web of Science, and Cochrane Library identified fourteen relevant studies. These studies reported immunomodulatory changes, including increased production of anti-inflammatory cells and cytokines. In mouse models of colitis treated with on helminth-derived proteins, significant improvements in pathological parameters such as body weight, colon length, and microscopic inflammatory scores were observed compared to control groups. Moreover, helminth-derived proteins can enhance the function of Tregs and alleviate the severity of inflammatory conditions. The findings underscore the pivotal role of helminth-derived proteins in immunomodulation, specifically in the axis of cytokine secretion and immune cell polarization. The findings offer new opportunities for treating chronic inflammatory conditions such Crohn’s disease.

## Introduction

Helminthes infections pose a significant global public health challenge, with recent estimations indicating that approximately 1.5 billion individuals are affected by one or more prevalent helminth infections. The majority of affected individuals reside in low- and middle-income countries located in endemic regions such as Asia, Latin America, the Caribbean, and sub-Saharan Africa [1]. These helminthes show notable variations in their biological life cycles and distinct variation in tissue tropism. The observed variations in clinical outcomes among helminth parasites can be attributed to these differences. The adverse outcomes of helminth infection are commonly linked to two factors: the level of parasite presence (also known as intensity or burden) and the duration of the infection, whether it is acute or chronic. Parasitic infection burden in humans have been linked to genetic phenotypes [1]. An initial genomic analysis of helminthes infection discovered a significant correlation between the intensity of parasitic infection and specific genetic markers encoding Th2 cytokines. The ongoing research effort in the field is exploring the host genes that have an impact on helminthes infection mostly focuses on schistosomiasis [2].

The duration of infection is a second contributing factor to the adverse outcome of helminthes infection. Numerous helminthiases are characterized by their chronic nature, and the mechanisms underlying the regulation of the Th2 immune response during the prolonged duration of these infections remains unclear, wherein antigen levels stay elevated and immunological-mediated pathology persists [3] There is a significant attention directed on the potential involvement of regulatory T cells (Tregs) in these physiological mechanisms,

potentially in conjunction with immunoregulatory molecules derived from helminths [4].

Recent studies have highlighted the significance of understanding the various mechanisms that parasites employ to modulate the immune response in favor of their survival within the host [5, 6]. . A review by Maizels et al. 2016 has indicated that the presence of live stages of the parasites in the host body can result in immunosuppression, but treating these parasites can restore the immune response of the host [5]. Furthermore, extracellular vesicles, proteins, or metabolites released or expelled by the parasite may also directly activate signaling pathways in host cells. Thus, scientists and researchers are investigating the proteins and components released by these parasites, as well as their role in overriding the host’s immune system [7]. Over time, humans have developed a balance between various immune responses due to childhood exposure to pathogens. The Th1/Th17 response is directed towards bacterial infections and autoimmunity, while the Th2 response is geared towards allergies and parasites. Parasitic infections can inhibit autoimmunity by promoting the Th2 response and inhibiting the Th1 response [8]. Research suggests that specific parasites may prevent allergic diseases and autoimmune disorders like multiple sclerosis, type 1 diabetes mellitus, and inflammatory bowel disease (IBD) [9].

IBD most often affects young adults, with symptoms ranging from abdominal pain, diarrhea, and bloody stool due to undetermined intestinal inflammation. There are two main types of IBD: Ulcerative Colitis (UC) and Crohn’s Disease (CD) [10]. These conditions affect the lining of the colon and the entire digestive tract, but the exact cause of IBD is still unknown. It is believed that an abnormal immune response to the bacteria in the gut may be responsible for the development of UC and CD [10]. Researchers have used various mouse models to study IBD and understand the mechanisms of chronic inflammation in the intestine. Chemicals such as Dextran Sodium Sulfate (DSS), Trinitrobenzene Sulfonic Acid (TNBS), and Dinitrobenzene Sulfonic Acid (DNBS) can be used to induce colitis, as well as spontaneous mutations, adoptive T-cell transfer, microbiome induction, and genetic engineering methods like IL-10 knockout mice [8, 11–14].

The management of inflammation is frequently achieved through the use anti-inflammatory medications, including corticosteroids, aminosalicylates, immunosuppressors, and anti-tumor necrosis factor (anti-TNF) agents. Surgical treatments are commonly advised as a treatment approach for cases of intestinal stenosis and fistula [8]. .Nevertheless, inflammatory bowel disease (IBD) is characterized by its resistance to treatment and the absence of a complete cure.

Given the immune-regulating properties of helminth infections, clinical trials have shown promising data using the helminth species *Trichuris suis* and *Necator americanus* in treating several inflammatory and autoimmune diseases [15]. Though, phase 2 trials of *Trichuris suis* failed to reach their clinical endpoints in IBD and multiple sclerosis as the effects of its treatment were modest in magnitude and varied greatly between study subjects. Furthermore, human helminth treatment should not be undertaken outside of controlled clinical trials [16–18]. The exploration and development of new medicinal substances derived from helminths are of paramount importance, given their well-documented anti-inflammatory properties. Our research efforts were focused on investigating the efficacy of helminth-derived proteins (HDPs) in animal (mouse) models of colitis.

## Methods

### Search strategy and study selection

The systematic review of Observational Studies in Epidemiology (MOOSE) guidelines [19]. It was also reported following the Preferred Reporting Items for Systematic Reviews (PRISMA) statement [20].

**Fig. 1:**
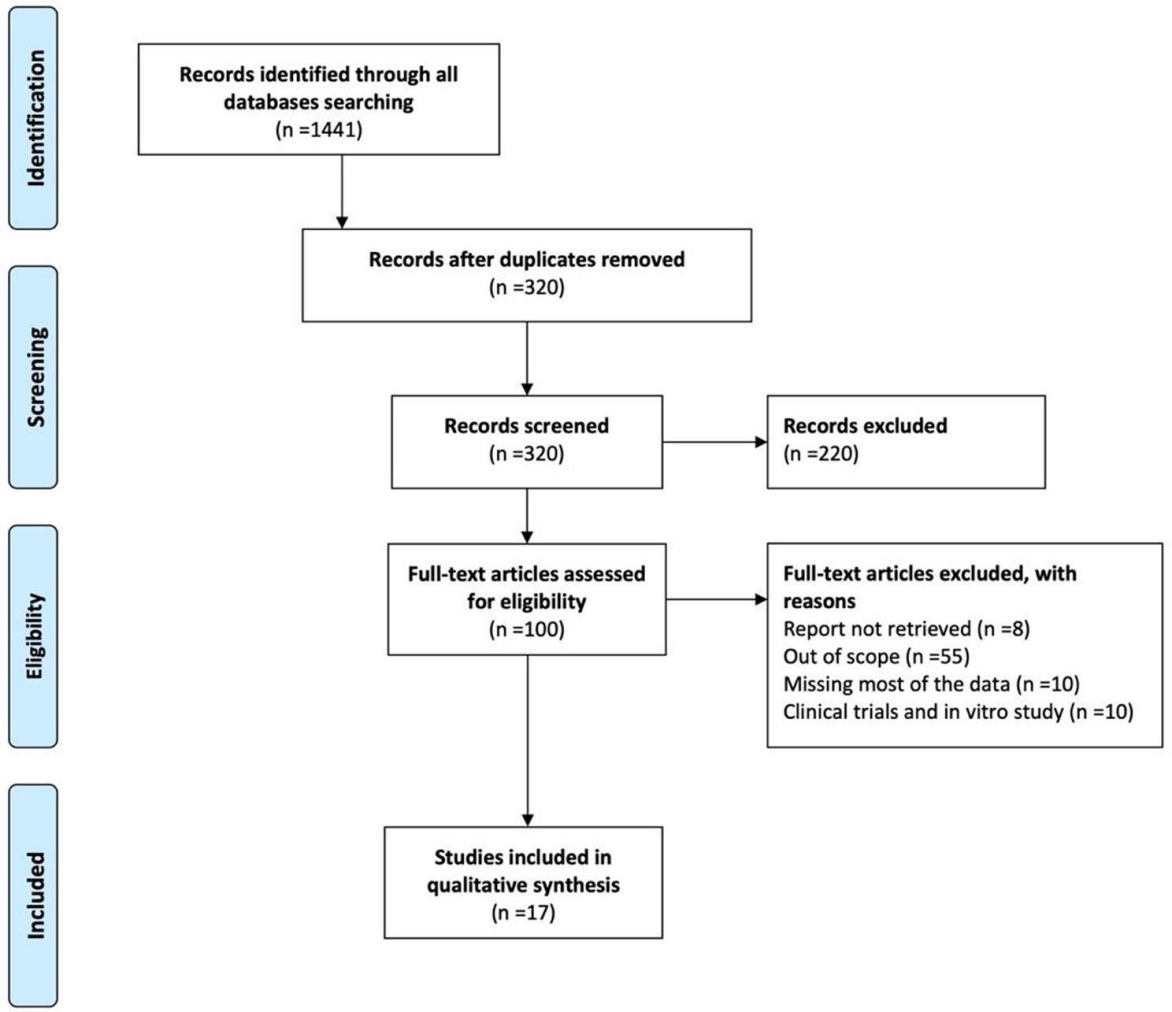
PRISMA Flow Diagram for Study Inclusion and Exclusion Process Using Multiple Databases. The databases used are Medline, EMBASE, Web of Science, and Cochrane Library databases. *Title and abstract screening are conducted to examine records. n = number of studies

To identify relevant studies, an extensive search approach was designed, as shown in Fig. 1. From inception until December 2022, a thorough electronic search of bibliographic databases such as Medline, Embase, and Cochrane Library was conducted. With English and American spellings, keywords, Emtree, and MeSH terms were employed. The following keywords were used in the search strategy: (“inflammatory bowel disease” OR “IBD” OR “Ulcerative colitis” OR “Crohn’s disease”) and (“parasites” OR “helminths” OR “worms” OR “parasite secretory products” OR “parasite products” OR “parasite-derived proteins”).

### Inclusion and exclusion criteria

We gathered studies investigating helminths’ immunomodulatory effects on colitis mouse models. We excluded conference proceedings, reviews, studies not published in English, in vitro studies, and clinical trials. Additionally, we excluded studies that utilized parasite infection or excretory-secretory (ES) methods for analysis.

Initially, AN and HA perform the search strategy for possible keywords that belong to the aim of this project. Then, the selected papers were screened by MA, AZ, TT, and FM, each study’s title and abstract evaluated independently to see if it meets the inclusion/exclusion criteria. As a result, all of the researchers (MA, AZ, TT, and FM) independently conducted a two-step full-text literature search to assess eligibility further.

### Data extraction and data synthesis

All researchers performed data extraction from the relevant studies independently using a standardized data collection form for all included studies. Each study retrieved the following demographic information: study description, data source, and type of helminths, type of colitis models, type of intervention, and compared groups. Moreover, the outcomes (cellular polarization, cytokines profile and pathological changes) were extracted from each study.

We categorized the helminths according to the external and internal morphology of egg, larval, and adult stages to trematodes, nematodes, filarial nematodes, and cestodes. Moreover, our data summarized the different IBD mouse models used by reported studies to induce colitis chemically (DSS, TNBS, or DNBS) and adoptive T Cell Transfer. In addition, Finally, we extracted the cellular and cytokines and pathological changes from each study to investigate the intervention changes compared to the control group. To assess the quality of included studies, we used the Systematic Review Center for Laboratory Animal Experimentation (the SYRCLE risk of bias tool) [21].

## Results

### Characteristics of included studies

From the original search we retrieved 1441 studies, of these, a total of 14 studies were thoroughly analysed as they met strict inclusion criteria (Fig. 1), focusing on proteins derived from helminths. The selected studies were conducted between the year of 2002 to the year of 2022. Our examination revealed that Schistosoma and Trichinella were the most extensively studied helminths, with six and four studies, respectively. Two studies were conducted on hookworms (*Necator americanus* and *Ancylostoma caninum*) and filarial nematodes (*Brugia malayi* and *Wuchereria bancrofti*). Additionally, one study was conducted on *Ascaris* and *Clonorchis*.

Among the Colitis-mouse studies, the TNBS-induced colitis model was the most frequently used, with eleven studies. Six studies employed the DSS-induced colitis model, while one utilized Adoptive T-cell transfer. Most studies compared the effect of each intervention to PBS, with only three studies adopting a different approach, as shown in Table 1.

**Table 1:** The demographic data of all the studies included in this systematic review

Parasite	Intervention	Animal model	Comparison	Reference
*Brugia malayi*	rBmCys	DSS-induced colitis mice	PBS	[22]
*Wuchereria bancrofti*	rWbL2	DSS-induced colitis	Aluminum sulfate.	[23]
*Schistosome Spp.*	P28GST	TNBS-induced colitis in rats and mice	Ethanol solution	[24]
*Schistosoma japonicum*	rSjcystatin	TNBS-induced colitis in mice	PBS	[13]
*Schistosoma japonicum*	rSj16	DSS-induced colitis mice	PBS	[25]
*Schistosoma haematobium*	P28GST	TNBS-induced colitis mice	Vehicle-treated	[26]
*Ancylostoma caninum*	(AIP)-1 and AIP-1_Q119_	TNBS-induced colitis in mice	PBS	[27]
*Necator americanus*	Na-AIP-1	TNBS-induced colitis mice	PBS	[28]
*Necator americanus*	Nak1	TNBS-induced colitis mice	Vehicle-treated	[29]
*Ancylostoma caninum*	Acan1	TNBS-induced colitis mice	Vehicle-treated	[29]
*Ascaris lumbricoides*	rAl-CPI	DSS-induced colitis mice	PBS	[30]
*Trichinella spiralis*	rTsP53	TNBS-induced colitis mice	Untreated	[31]
*Trichinella spiralis*	NBL-SP	TNBS-induced colitis	PBS	[32]
*Trichinella spiralis*	TsKaSPI, TsAdSPI	TNBS-induced colitis	PBS	[33]
*Trichinella spiralis*	rTsPmy	DSS-induced colitis	PBS	[34]
*Clonorchis sinensis*	CsStefin-1	DSS-induced colitis	PBS	[35]

*Abbreviations* rBmCys (*Brugia malayi* recombinant cystatin), rWbL2 (*Wuchereria bancrofti* larval recombinant protein), P28GST (Schistosome 28-kDa glutathione S-transferase), rSjcystatin (*Schistosoma japonicum* recombinant cystatin), rSj16 (*S. japonicum* recombinant 16-kDa secreted protein), (AIP)-1(*Ancylostoma caninum* recombinant protein anti-inflammatory protein-1), AIP-1_Q119_ (the mutant recombinant protein AIP-1), Na-AIP-1 (*Necator americanus* Netrin domain-containing proteins), Nak1 (*N.americanus* low-MW peptide found in the excretory/secretory proteins), Acan1 (*A. caninum* low-MW peptides (∼4 kDa each) found in the excretory/secretory proteins), rAl-CPI (*Ascaris lumbricoides* recombinant cystatin protease), rTsP53 (*Trichinella spiralis* recombinant 53-kDa protein), NBL-SP (*T. spiralis* newborn larvae recombinant serine protease), TsKaSPI (*T. spiralis* Kazal-type serine protease inhibitors recombinant protein ), TsAdSPI (*T.spiralis* adult serine protease inhibitors recombinant protein), rTsPmy (*T. spiralis* paramyosin recombinant protein), CsStefin-1 (*Clonorchis sinensis* Type I cystatin recombinant protein)

### Quality of included studies

**Fig. 2:**
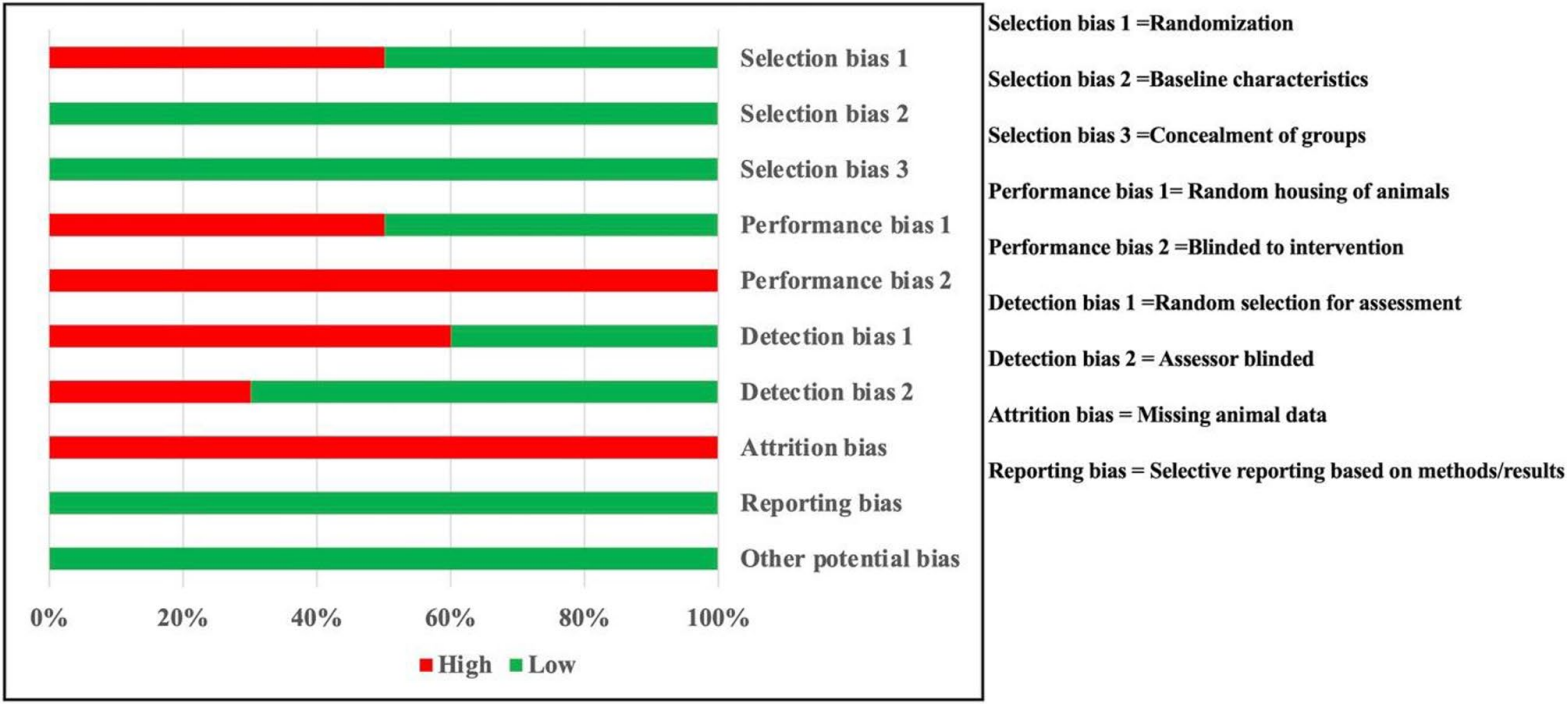
Risk-of-bias assessment graph, using the SYRCLE tool: review authors’ judgments for each domain; Selection, Performance, Detection, Attrition, Reporting, and Other Bias presented as percentages across the included studies

Based on a comprehensive analysis of animal studies using the SYRCLE risk of bias tool, we have identified specific categories that display a higher to intermediate risk of bias, including randomization, performance, detection, and attribution. However, we have also come across categories indicating a low risk of bias, such as selection, reporting, and potential biases. Please refer to Fig. 2 and Table S1 for a more detailed understanding.

### The immunoregulatory effect of recombinant proteins derived from helminths

Polarization of immune cells is a common feature of the host response to microbial infection, inflammation, fibrosis, and tissue repair. Different studies illustrated the ability of parasites to modify hosts’ immune systems and contribute to the polarization of their immune response. We have found that several studies suggest that helminths could positively impact the immune system by increasing the number of regulatory T cells (Table 2). Although these studies used different approaches to investigate the effects of various helminths on colitis, they have shown that proteins from various helminths can boost Treg cell expansion in critical areas of the body, including the mLN, Lamina propria, Spleen, and colon.

**Table 2:** The effect of helminth-drive protein intervention on T regulatory cell polarization in the IBD-mouse model compared to the control group

Parasite	Parasite product	T cells polarization	Reference
*Schistosoma japonicum*	rSjcystatin	↑ CD4+ CD25+ FOXP3+ Treg	[13]
*Schistosoma japonicum*	rSj16	↑ CD4+ CD25+ FOXP3+ Treg	[25]
*Ancylostoma caninum*	AIP-1 and AIP-1_Q119_	↑ CD4+ CD25+ FOXP3+ Treg	[27]
*Trichinella spiralis*	NBL-SP	↑ CD4+ CD25+ FOXP3+ Treg	[32]
*Trichinella spiralis*	TsKaSPI, TsAdSPI	↑ CD4+ CD25+ FOXP3+ Treg	[33]
*Trichinella spiralis*	rTsPmy	↑ CD4+ CD25+ FOXP3+ Treg	[34]
*Clonorchis sinensis*	CsStefin-1	↑ CD4+ CD25+ FOXP3+ Treg	[35]

*Abbreviations* ND: Not detected, ↑ increase, ↓ decrease. rBmCys (*Brugia malayi* recombinant cystatin), rWbL2 (*Wuchereria bancrofti* larval recombinant protein), P28GST (Schistosome 28-kDa glutathione S-transferase), rSjcystatin (*Schistosoma japonicum* recombinant cystatin), rSj16 (*S. japonicum* recombinant 16-kDa secreted protein), (AIP)-1(*Ancylostoma caninum* recombinant protein anti-inflammatory protein-1), AIP-1_Q119_ (the mutant recombinant protein AIP-1), Na-AIP-1 (*Necator americanus* Netrin domain-containing proteins), Nak1 (*N.americanus* low-MW peptide found in the excretory/secretory proteins), Acan1 (*A. caninum* low-MW peptides (↑4 kDa each) found in the excretory/secretory proteins), rAl-CPI (*Ascaris lumbricoides* recombinant cystatin protease), rTsP53 (*Trichinella spiralis* recombinant 53-kDa protein), NBL-SP (*T. spiralis* newborn larvae recombinant serine protease), TsKaSPI (*T. spiralis* Kazal-type serine protease inhibitors recombinant protein ), TsAdSPI (*T. spiralis* adult serine protease inhibitors recombinant protein), rTsPmy (*T. spiralis* paramyosin recombinant protein), CsStefin-1 (*Clonorchis sinensis* Type I cystatin recombinant protein)

When proteins from helminths are administered to mice with induced colitis, it reduces the production of Th1/Th17 cytokines, such as IL-1β, IL-6, TNF-α, IL-17, and IFN-γ, while increasing the production of Th2/Treg cytokines, such as IL-4, IL-13, TGFβ, and IL-10 in the serum, colon homogenates, and spleen (< Emphasis Type="Bold”> Table 3</Emphasis> ). After analyzing the colon homogenates cytokine profile following the treatment with helminths-derived renominate protein, there was a significant decrease in IL-1β, IL-6, TNF-α, IL-17, and IFN-γ levels and an increase in the levels of IL-4, IL-13, TGFβ, and IL-10. However, a study revealed that treatment with Trichinella spiralis-derived recombinant protein leads to a rise in TNF-α and IFN-γ in serum.

Regarding serum, most studies showed similar cytokine profiling after treatment with helminths-derived renominate protein to colon homogenates. However, one study demonstrated an increase in pro-inflammatory cytokines. Finally, cytokine changes from mesenteric lymph nodes (mLN) and spleen showed a similar pattern.

**Table 3:** The effect of helminth-drive protein intervention on cytokine changes in the IBD-mouse model compared to control group

Parasite	Parasite product	Colon homogenate (Th1)	Colon homogenate (Th2)	Reference
*Schistosome cercariae*	P28GST	↓ IL-1β, ↓ TNF-α, ↓ IL-17A	↑IL-10, ↑IL-13	[24]
*Schisotosoma japonicum*	rSjcystatin	↑IL-4, ↓IFN-γ	↑TGFβ, ↑IL-10, ↑ IL-13	[13]
*Schistosoma japonicum*	rSj16	↓IL-6, ↓ IL-1β, ↓TNF-α, ↓ IL-17A, ↓IFN-γ,	↑TGFβ, ↑IL-10	[25]
*Ancylostoma caninum*	AIP-1 and AIP-1_Q119_	↓TNF-α, ↓ IL-17A, ↓IFN-γ	↑IL-10, ↑TGFβ	[27]
*Ascaris lumbricoides*	rAl-CPI	↓IL-6, ↓ IL-1β, ↓TNF-α, ↓IFN-γ,	↑TGFβ, ↑IL-10	[30]
*Trichinella spiralis*	rTsP53	↓TNF-α, ↓ IL-6	↑IL10 ↑TGFβ	[31]
*Trichinella spiralis*	NBL-SP	↓IFN- γ, ↓ IL-17	ND	[32]
*Trichinella spiralis*	TsKaSPI, TsAdSPI	↓IFN-γ	↑IL-4	[33]
*Trichinella spiralis*	rTsPmy	↓TNF-α ,↓ IL-1β, ↓IL-17, ↓IL-6, ↓IFN-γ	↑IL-10, ↑TGFβ	[34]
**Parasite**	**Parasite product**	**Serum (Th1)**	**Serum (Th2)**	**Reference**
*Brugia malayi*	rBmCys	↓TNF-α, ↓IFN-γ	↑IL-10	[22]
*Schistosoma mansoni*	SmSWP	↓TNF- α, ↓ IL-6, ↓IL-17a, ↓IFN-γ	↑ IL-4, ↑ IL-10	[37]
*Schistosoma haematobium*	P28GST	↑TNF-α,↓ IL-1β, ↓IL-6	ND	[26]
*Trichinella spiralis*	rTsP53	↓TNF-α, ↓IFN-γ	↑IL-13, ↑IL-4	[31]
*Trichinella spiralis*	NBL-SP	↓IFN- γ,↓TNF, ↓IL-17	↑IL-4, ↑IL-10, ↑TGF-β	[32]
**Parasite**	**Parasite product**	**MLN or spleen (Th1)**	**MLN or spleen (Th2)**	**Reference**
*Brugia malayi*	rBmCys	↓IL-6, ↓TNF-α, ↓ IL-17A, ↓IFN-γ	↑IL-10	[22]
*Wuchereria bancrofti*	rWbL2	↓IL-6, ↓TNF-α, ↓ IL-17A, ↓IFN-γ	↑IL-10	[23]
*Schistosome cercariae*	P28GST	↓ IL-1β, ↓TNF-α, ↓ IL-17A, ↓IFN-γ	↑IL-10, ↑IL-13	[24]
*Schisotosoma japonicum*	rSjcystatin	↓IFN-γ, ↓IL-17A	↑TGF-β, ↑IL-10	[13]
*Schistosoma mansoni*	SmSWP	↓IFN-γ, ↓ IL-17	↑TGF-β, ↑IL-10	[38]
Ascaris lumbricoides	rAl-CPI	↓IFN-γ	↑TGF-β, ↑IL-10	[30]
*Trichinella spiralis*	TsKaSPI, TsAdSPI	↓IFN- γ	↑IL-4	[33]
*Clonorchis sinensis*	CsStefin-1	↓ TNF-α, ↓FN-γ	↑ IL-10, ↑TGF-β	[35]

*Abbreviations* ND: Not detected, ↑ increase, ↓ decrease. rBmCys (*Brugia malayi* recombinant cystatin), rWbL2 (*Wuchereria bancrofti* larval recombinant protein), P28GST (Schistosome 28-kDa glutathione S-transferase), rSjcystatin (*Schistosoma japonicum* recombinant cystatin), rSj16 (*S. japonicum* recombinant 16-kDa secreted protein), (AIP)-1(*Ancylostoma caninum* recombinant protein anti-inflammatory protein-1), AIP-1_Q119_ (the mutant recombinant protein AIP-1), Na-AIP-1 (*Necator americanus* Netrin domain-containing proteins), Nak1 (*N.americanus* low-MW peptide found in the excretory/secretory proteins), Acan1 (*A. caninum* low-MW peptides (∼4 kDa each) found in the excretory/secretory proteins), rAl-CPI (*Ascaris lumbricoides* recombinant cystatin protease), rTsP53 (*Trichinella spiralis* recombinant 53-kDa protein), NBL-SP (*T. spiralis* newborn larvae recombinant serine protease), TsKaSPI (*T. spiralis* Kazal-type serine protease inhibitors recombinant protein ), TsAdSPI (*T. spiralis* adult serine protease inhibitors recombinant protein), rTsPmy (*T. spiralis* paramyosin recombinant protein), CsStefin-1 (*Clonorchis sinensis* Type I cystatin recombinant protein)

### Macroscopic/microscopic inflammatory score and disease activity index score

Based on the findings presented in Table 4, recombinant proteins sourced from helminths have demonstrated efficacy in reducing inflammation in mice with colitis. This mode of treatment has correspondingly yielded improvements in colon length and body weight while reducing both DAI and microscopic inflammatory scores. Furthermore, these studies have shown decreased myeloperoxidase activity (MPO).

**Table 4:** The effect of helminth-drive protein intervention on pathological changes in the IBD-mouse model compared to the control group

Parasite	Parasite product	Body weight	Colon length	Disease activity index (DAI)	Myeloperoxidase (MPO) activity	Microscopic score/histological/ inflammation score	Macroscopic score	Reference
*Brugia malayi*	rBmCys	↑	↑	↓	↓	↓	ND	[22]
*Wuchereria bancrofti*	rWbL2	↑	↑	↓	ND	ND	ND	[23]
*Schistosome cercariae*	P28GST	↓	↑	ND	↓	↓	ND	[24]
*Schisotosoma japonicum*	rSjcystatin	↑	↑	↓	↓	↓	↓	[13]
*Schistosoma japonicum*	rSj16	↑	↑	↓	↓	↓	↓	[25]
*Schistosoma haematobium*	P28GST	↑	ND	↓	↓	↓	↓	[26]
*Ancylostoma caninum*	AIP-1 and AIP-1_Q119_	↑	↑	ND	ND	↓	↓	[27]
*Necator americanus*	Na-AIP-1	↑	↑	ND	ND	↓	↓	[28]
*Necator americanus*	Nak1	↑	↑	ND	ND	ND	ND	[29]
*Ancylostoma caninum*	Acan1	↑	↑	ND	ND	ND	ND	[29]
*Ascaris lumbricoides*	rAl-CPI	↑	↑	↓	↓	↓	ND	[30]
*Trichinella spiralis*	rTsP53	↑	ND	↓	ND	↓	↓	[31]
*Trichinella spiralis*	NBL-SP	ND	↑	↑	ND	↓	↓	[32]
*Trichinella spiralis*	TsKaSPI, TsAdSPI	↓	ND	↓	↓	↓	↓	[33]
*Trichinella spiralis*	rTsPmy	↑	↑	ND	ND	↓	↓	[34]
*Clonorchis sinensis*	CsStefin-1	↑	↑	↓	ND	↓	↓	[35]

## Discussion

Exploring how parasites adapt to their host environments can have significant therapeutic implications in treating immune-mediated diseases. Research into helminth parasites and their bioproducts can reduce inflammation associated with inflammatory bowel disease (IBD).

Several studies exploited the effects of helminth-derived proteins and peptides on different autoimmune and inflammatory disorders, and most of them showed the ability of these components to regulate the immune response and improve the pathology of these disorders. Moreover, some of these proteins and peptides were studied in vaccine development and showed promising results. However, to our knowledge, all these studies were in the preclinical phase and yet not in clinical trials [36].

**Fig. 3:**
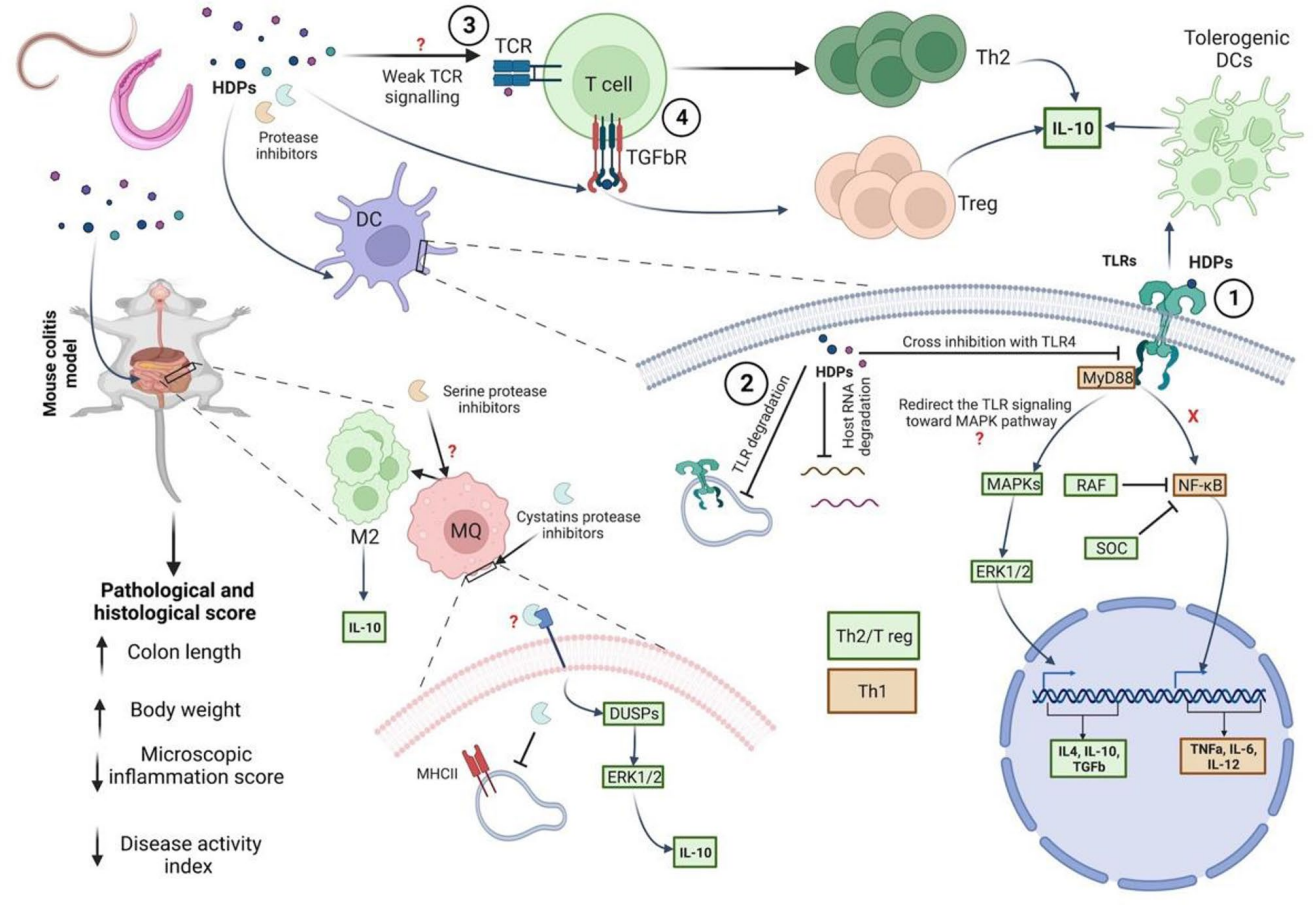
Helminth-derived proteins (HDPs) manipulate the host’s immune system to favour the parasite by engaging receptors, degrading molecules, interfering with signals, and mimicking host cytokines. (**1**) Certain components of HDPs can redirect immune responses towards anti-inflammatory and Th2 responses by priming Th2/Treg-inducing DCs. (**2**) HDPs may manipulate the host’s immune system through intracellular molecule degradation, such as breaking down TLR3, disrupting inflammation pathways, suppressing protein synthesis, and causing Th2 polarization. (**3**) Certain proteins in helminths can suppress the immune system by interfering with signals, such as interfering with T cell receptors and MHCII. (**4**) Certain HDPs could mimic cytokines and convert naïve T cells into regulatory T cells. Created with BioRender.com

Our primary objective is to assess the effectiveness of HDPs with colitis-induced models. Our findings suggest that HDPs derived from intestinal nematodes, filarial nematodes and trematodes, have the potential to modulate the immune response in these models. This regulation is accomplished by activating regulatory T cells, utilizing regulatory cytokines such as IL-10, and enhancing the Th2 immune response while diminishing the Th1 response. It has been previously shown that Tregs can play an important role in regulating immune responses and maintaining homeostasis in several diseases including autoimmune and inflammatory diseases either through cell-cell contact or through the production of immunosuppressive cytokines eg, IL-10 and TGF- β. For instance, type 1 Tregs cells are known to secrete high levels of IL-10, whereas type 3 Tregs cells are recognized for their TGF-β secretion [15]. HDPs are believed to modulate the host’s immune response in favour of the parasite. Immune manipulation mechanisms by HDPs are many some examples includes engaging immune cell receptors, breaking down molecules, disrupting cell-cell signals, and mimicking cytokines (Fig. 3). A remarkable example of how HDPs modulate the host immune response is observing the outcome of the inoculation of hookworms-derived antigens, which are a complex mixture of over 100 proteins with unknown functions in colitis-induced mouse models resulted in the induction of great levels of T-helper type 2 (Th2-related) cells and regulatory cytokines, including IL-10, IL-4, and TGF-β and a corresponding decrease in the inflammatory cytokines including IL-13, TNF-β, IL-1, IFN-γ, and IL-17a [39–42]. Moreover, IBD-related symptoms have been improved after hookworm-derived antigens inoculation. Other studies also highlight that selected hookworm-derived products could be used to treat autoimmune disorders without using whole live parasites. For example, the most abundant protein produced by hookworm, anti-inflammatory protein (AIP)-1, promoted the recruitment of Treg cells to the gut and allowed rapid healing of the colon mucosa, which aids in the maintenance of the intestine’s tolerance state [42–44]. Hookworms have also been shown to induce a type 2 immune response associated with increased circulatory eosinophils[45].

HDPs exhibit immunoregulatory characteristics. For instance, the tapeworm *Echinococcus granulosus* inhibits the maturation of DCs, and the differentiation of monocytes, resulting in a shift towards a regulated immune [44]. Interestingly, HDPs can also regulate Toll-like receptors (TLRs) signaling to induce a state of tolerance in DCs which subsequently induces the production of anti-inflammatory cytokines such as IL-10 [45]. IL-10 exerts immunosuppressive effects by directly inhibiting TLR signaling, resulting in the overall suppression of APCs activation, and thus inhibiting MHC class II expression. Several mechanisms have been suggested to explain the reduction of APC function by IL-10, The intracellular pathways targeted by these biomolecules are primarily undefined. TLRs are the most described mechanism of their immunomodulatory effect. Interestingly, HDPs can also regulate TLRs signaling to produce a tolerogenic dendritic cell phenotype that secretes anti-inflammatory cytokines [46].

*Schistosoma mansoni* and its eggs can both produce bioactive antigens, including lysophosphatidylserine, and lacto-N-fucopentaose III (LNFPIII), which can alleviate inflammatory responses by targeting TLRs [47]. It has been found that simultaneously and non-simultaneously activating TLR by HDPs can activate intracellular signaling pathways. This activation is critical in suppressing a Th1 response and polarizing immune responses towards anti-inflammatory and Th2 responses. Examples include *Trichinella spiralis* muscle larvae, *Schistosoma mansoni*, and *Ascaris lumbricoides*-derived phospholipids. These components induce transient ERK1/2 signaling, priming Th2/Treg-inducing DCs and supporting Th2 polarization by generating an imbalance in TLR2 signaling and strengthening the ERK pathway in human monocyte-derived DCs [48, 49]. It is also worth noting that different stages of nematodes have been demonstrated to inhibit NF-κB translocation and phosphorylation of p38 and ERK1/2 in LPS-treated murine macrophage cell lines [50]. Additionally, HDP from *Schistosoma mansoni* can enter the endosome and degrade TLR3, disrupting intracellular pathways activated during inflammation [51]. Moreover, the suppression of protein synthesis and subsequent Th2 polarization is attributed to the omega-1 protein obtained from Schistosomes [52].

Helminths, like filarial nematodes, nematodes, and trematodes, contain protease inhibitors such as serine and cystatins in their excretory-secretory products. Macrophages uptake filarial cystatin protease inhibitors, which target ERK1/2 and p38 by stimulating dual specificity phosphatases (DUSPs), negative MAPK signalling and IL-10 expression regulators. This helps to modulate downstream signals, inducing regulatory responses [53]. *Ascaris lumbricoides* cysteine protease inhibitors regulate the immune system by suppressing MHC-II expression and dendritic cell-mediated antigen presentation while promoting nitric oxide and regulatory cytokines like IL-10 and TGF-β. It also helps regulate macrophages for further immune response benefits [54–58]. During the early stages of *T. spiralis* infection, the secretion of serine protease inhibitors plays a crucial role in immunosuppression. The serine protease inhibitors directly activate macrophages with an alternatively activated phenotype, which helps the adult worms survive in the intestinal tract [59]. Furthermore, Parasite-derived cystatins, such as CPI2 from *B. malayi* and onchocystatin from *Onchocerca volvulus*, directly interfere with antigen presentation. This interference occurs by inhibiting proteases involved in antigen presentation, which prevents T-cell activation at the level of T-cell receptor engagement by MHC complexes. Additionally, these cystatins enhance the production of interleukin-10 (IL-10) by APCs [60].

On the other hand, it has been observed that specific proteins obtained from helminths can efficiently weaken the immune system without the involvement of Pattern Recognition Receptors (PRRs) such as TLR. This can lead to interference with antigen presentation, disturbance in T cell receptor (TCR) signalling and mimicking or inhibiting host cytokines.

Low-dose HPDs may prompt weak TCR signaling, resulting in a Th2 response. This hypothesis is based on a study that established a correlation between antigen dosage, cytokine levels, and Th cell characteristics [61]. Additionally, TGF-β homologs have been found in nematodes and trematodes. This highly potent cytokine has immunoregulatory properties that stimulate the transformation of naïve T cells into regulatory T cells [62].

One of the study’s limitation is its focus on mouse models of colitis, which may not accurately represent human immune responses. Additional research involving human subjects is necessary to validate the therapeutic potential of helminth-derived proteins.

Moreover, investigating the impact of proteins obtained from various sources of helminths and taking into account the complex interactions between host immune systems and different species of helminths should improve the applicability of the results. Future studies may need to explore the effects of intact helminths or their various components for a comprehensive understanding of the immunomodulatory potential of helminths

## Conclusion

The impact of HDPs on mice with colitis was investigated. The findings indicate that HDPs can regulate the immune system by augmenting the production of anti-inflammatory cytokines and diminishing pro-inflammatory cytokines locally and systemically. Additionally, HDPs can enhance the function of Tregs and alleviate the severity of inflammatory conditions. Additional investigation is needed in order to achieve a comprehensive understanding of the mechanisms by which HDPs enhance the management of inflammatory bowel disease (IBD) and facilitate the creation of innovative therapies for chronic inflammatory conditions.

## Data Availability

No datasets were generated or analysed during the current study.

## Abbreviations

ND: Not detected, ↑ increase, ↓ decrease
rBmCys: (*Brugia malayi* recombinant cystatin)
rWbL2: (*Wuchereria bancrofti* larval recombinant protein)
SmSWP: (*Schistosoma mansoni* soluble worm proteins)
P28GST: (Schistosome 28-kDa glutathione S-transferase)
rSjcystatin: (*Schistosoma japonicum* recombinant cystatin)
rSj16: *(S. japonicum* recombinant 16-kDa secreted protein)
(AIP)-1: (*Ancylostoma caninum* recombinant protein anti-inflammatory protein-1)
AIP-1Q119: (the mutant recombinant protein AIP-1)
Na-AIP-1: (*Necator americanus* Netrin domain-containing proteins)
Nak1: (*N.americanus* low-MW peptide found in the excretory/secretory proteins)
Acan1: (*Ancylostoma caninum* recombinant protein anti-inflammatory protein-1)
(AIP)-1: (*A. caninum* low-MW peptides (∼4 kDa each) found in the excretory/secretory proteins)
rAl-CPI: (*Ascaris lumbricoides* recombinant cystatin protease)
rTsP53: (*Trichinella spiralis* recombinant 53-kDa protein)
NBL-SP: (*T. spiralis* newborn larvae recombinant serine protease)
TsKaSPI: (*T. spiralis* Kazal-type serine protease inhibitors recombinant protein )
TsAdSPI: (*T. spiralis* adult serine protease inhibitors recombinant protein)
rTsPmy: (*T. spiralis* paramyosin recombinant protein)
CsStefin-1: (*Clonorchis sinensis* Type I cystatin recombinant protein)
