# Correction: Helminth-derived proteins as immune system regulators: a systematic review of their promise in alleviating colitis

**DOI:** 10.1186/s12865-024-00661-9

**Published:** 2024-10-04

**Authors:** Maimonah Alghanmi, Faisal Minshawi, Tarfa A. Altorki, Ayat Zawawi, Isra Alsaady, Abdallah Y. Naser, Hassan Alwafi, Soa’ad M. Alsulami, Ala A. Azhari, Anwar M. Hashem, Rowa Alhabbab

**Affiliations:** 1 Department of Medical Laboratory Sciences, Faculty of Applied Medical Sciences, King Abdulaziz University, Jeddah, Saudi Arabia; 2 Vaccines and Immunotherapy Unit, King Fahd Medical Research Center, King Abdulaziz University, Jeddah, Saudi Arabia; 3 Department of Laboratory Medicine, Faculty of Applied Medical Sciences, Umm Al-Qura University, Makkah, Saudi Arabia; 4 Special Infectious Agent Unit, King Fahad Medical Research Center, King Abdulaziz University, Jeddah, Saudi Arabia; 5 Department of Applied Pharmaceutical Sciences and Clinical Pharmacy, Faculty of Pharmacy, Isra University, Amman, Jordan; 6 Faculty of Medicine, Umm Al-Qura University, Makkah, Saudi Arabia; 7 Clinical and Molecular Microbiology Laboratories, King Abdulaziz University Hospital, King Abdulaziz University, Jeddah, 21589 Saudi Arabia; 8 Department of Clinical Microbiology and Immunology, Faculty of Medicine, King Abdulaziz University, Jeddah, Saudi Arabia

**Correction: BMC Immunology 25, 21 (2024)**

**https://doi.org/10.1186/s12865-024-00614-2**

Following publication of the original article [1], an error was noticed in the grant number relating to the Institutional Fund Projects in the Funding section was incorrect.

Incorrect grant number

Institutional Fund Projects under grant no (IFPRC408160-290-2020)

Correct grant number

Institutional Fund Projects under grant no (IFPRC-045-142-2020)

The original article has been corrected.
